# Secondary Decomposers Meet Their Predators: Decomposition Stage and Substrate Quality Jointly Structure Microbial Brown Food Webs During Fungal Necromass Decay

**DOI:** 10.1111/mec.70060

**Published:** 2025-08-08

**Authors:** François Maillard, Briana H. Beatty, Stefan Geisen, Enrique Lara, Peter G. Kennedy

**Affiliations:** ^1^ Microbial Ecology, Department of Biology Lund University Lund Sweden; ^2^ Department of Plant and Microbial Biology University of Minnesota St. Paul Minnesota USA; ^3^ Laboratory of Nematology Wageningen University and Research Wageningen the Netherlands; ^4^ Real Jardín Botánico‐CSIC Madrid Spain

**Keywords:** fungal necromass, microbial predators, nematodes, protists, secondary decomposers

## Abstract

Mycelial residues, also known as fungal necromass, represent a substantial fraction of soil organic matter (SOM) pools in terrestrial ecosystems worldwide. Although microbial decomposers are increasingly recognised as key drivers of fungal necromass carbon stock formation, the diversity and composition of their microbial predators—and the roles these predators play in mediating fungal necromass decomposition—have not been explored to date. To address this gap, we produced fungal necromass of varying biochemical quality from *Hyaloscypha bicolor* and decomposed it in forest topsoil in Minnesota, USA, to investigate how microbial decomposer (bacteria and fungi) and predator (protists and nematodes) communities differ between soil and necromass. We also examined whether microbial predators influence the abundance of fungal necromass decomposers and affect necromass decomposition rates. Over two sampling times (4 and 12 weeks), necromass exhibited rapid early mass loss followed by reduced decay, with a higher stabilised mass in high melanin necromass. Microbial abundances were higher in necromass than in surrounding soil, especially in low melanin necromass. Community composition of both decomposers and their predators differed between soil and necromass and shifted markedly with necromass quality and decomposition stage. Predator community composition was linked to bacterial and fungal abundances at both early and late stages of decay and was marginally associated with decomposition rates. We conclude that fungal necromass acts as a microbial ‘hotspot’ not only for decomposers but also for their predators. These findings highlight the importance of microbial predator–decomposer interactions to better understand the formation of fungal‐derived SOM.

## 1 Introduction

Soil organic matter (SOM) constitutes the largest active reservoir of organic carbon in terrestrial ecosystems (Scharlemann et al. 2014), playing a critical role in atmospheric carbon capture and climate change mitigation (Lal 2004). Consequently, understanding the mechanisms that drive the formation and stabilisation of soil organic carbon (SOC) is of critical importance. Recent studies have shown that dead mycelial residues—termed fungal necromass—may account for more than 50% of the SOC pool in terrestrial ecosystems (Angst et al. 2021; Liang et al. 2019; Wang et al. 2021). These estimates are primarily based on amino sugar quantifications, which serve as reliable biomarkers for estimating SOC contributions from microbial necromass (Liang et al. 2019). Despite growing insight into the distribution and magnitude of fungal necromass carbon stocks (Li et al. 2022; Liu et al. 2024), the biochemical and ecological processes underpinning SOM formation from fungal sources remain poorly understood. In particular, the role of the ‘brown food web’—that is, the decomposer community and its predators—in transforming senesced mycelial necromass into stabilised SOM has yet to be elucidated (Camenzind et al. 2023; Sokol et al. 2022).

In temperate and boreal ecosystems, soil microbes drive SOM formation through their saprotrophic activities, which involve secreting extracellular enzymes to decompose organic residues and assimilate carbon for growth (Baldrian 2017; Kallenbach et al. 2016; Paul 2016; Schlesinger and Andrews 2000). During saprotrophy, carbon is either respired, assimilated into microbial biomass, or transformed into degradation byproducts that contribute to SOC stocks through biochemical recalcitrance, mineral adsorption, or physical occlusion (Cotrufo and Lavallee 2021; Liang et al. 2017; Lützow et al. 2006). However, decomposition processes vary among microbial decomposer groups due to functional differences. For instance, bacteria typically exhibit lower carbon use efficiency (CUE) compared to fungi, often resulting in lower SOC stabilisation (Allison et al. 2013; Kallenbach et al. 2016; Strickland and Rousk 2010). Additionally, fungi display diverse decomposition pathways through varied enzyme secretion profiles and non‐enzymatic degradation systems, leading to biochemically heterogeneous SOM affecting SOC persistence (Floudas et al. 2015; Maillard, Jusino, et al. 2022; Stutz et al. 2017; Zhang et al. 2016). As a consequence, microbial communities involved in decomposing plant residues—referred to as ‘primary decomposers’ by Morrissey et al. (2023)—have been extensively studied to inform soil carbon models (Wieder et al. 2015), particularly regarding the distinct roles of bacteria and fungi in the decomposition processes and their broader ecosystem consequences. In contrast, far less is known about ‘secondary decomposers’, a category introduced by Morrissey et al. (2023) that specifically targets microbial necromass.

Regarding fungal necromass decomposer community structure, there is growing evidence that a relatively conserved set of secondary decomposers participates in the decomposition processes, forming what is known as the fungal necrobiome (Beidler et al. 2020; Cantoran et al. 2023). Unlike plant litter decomposition, which is predominantly driven by fungi, fungal necromass decomposition engages both bacterial and fungal taxa (López‐Mondéjar et al. 2020; Maillard, Michaud, et al. 2023). For instance, a recent quantitative stable isotope probing (qSIP) study tracking necromass carbon into microbial DNA identified a diverse array of both bacterial and fungal taxa actively decomposing fungal necromass in situ (Maillard, Michaud, et al. 2023). Necromass‐associated bacterial communities are typically dominated by copiotrophic taxa, often from lineages highly efficient in degrading fungal polymers such as chitin (e.g., *Chitinophaga*, *Streptomyces*) (Beidler et al. 2020; Brabcová et al. 2016; Maillard et al. 2021). Similarly, necromass‐associated fungal communities are dominated by fast‐growing saprotrophic lineages within groups like Ascomycota and Mortierellomycota, as well as mycoparasitic fungi that likely function as facultative saprotrophs (Brabcová et al. 2016, 2018; Fernandez and Kennedy 2018; Maillard et al. 2020; Maillard, Michaud, et al. 2023). In forest ecosystems, plant‐associated fungi, such as ectomycorrhizal fungi, also colonise fungal necromass, particularly at later stages, likely to obtain nutrients rather than carbon (Akroume et al. 2019; Fernandez and Kennedy 2018; Maillard et al. 2021; Maillard, Fernandez, et al. 2022). Collectively, these studies demonstrate that secondary decomposers of fungal necromass exhibit both taxonomic and functional diversity.

Emerging evidence indicates that the composition of secondary decomposer communities can significantly impact fungal necromass decomposition rates. Recent studies have demonstrated that reducing ectomycorrhizal fungi in soils led to faster fungal necromass decomposition, suggesting that this guild may slow necromass decomposition and thereby contribute to increased SOC stocks of fungal origin (Beidler et al. 2021; DeLancey et al. 2024). Analyses utilising soil microbial communities as predictors for fungal necromass decay have also revealed that both soil bacterial richness and fungal community structure are significant predictors of the extent of fungal necromass mass loss (Beidler et al. 2025; Maillard, Beatty, et al. 2023). Moreover, correlative studies also indicate that specific fungal taxa can either accelerate or decelerate fungal necromass decomposition (Maillard et al. 2021; Maillard, Beatty, et al. 2023). These opposing effects reflect the functional diversity within microbial decomposer communities, resulting in varied organic matter decay rates (Pérez‐Pazos et al. 2024).

Research also suggests that secondary decomposers represent a specialised subset of the broader soil microbial communities, with a markedly different composition (Beidler et al. 2020; Brabcová et al. 2016). Indeed, Beidler et al. (2020) reported lower bacterial and fungal diversity on necromass compared to bulk soil. Further, Brabcová et al. (2016) observed bacterial abundances approximately eight times higher and fungal abundances two to three times higher in fungal necromass compared to bulk soil after 3 months of decomposition. These patterns are consistent with necromass acting as a resource‐rich ‘hotspot’ of carbon and nutrients for colonising secondary decomposers, relative to the more oligotrophic surrounding soil. Consequently, decomposing fungal necromass should be considered a nutrient‐rich, ephemeral resource patch (Butterworth et al. 2023), which likely functions as an environmental filter shaping soil decomposer communities. Therefore, identifying the microbial taxa inhabiting soils and participating in fungal necromass decomposition—along with understanding their respective effects on fungal necromass carbon formation—is crucial for assessing the extent to which specific soil microbial communities may promote or impede the formation of SOM of fungal origin. This is especially important given that global surveys of microbial diversity and composition have predominantly focused on bulk soil communities rather than decomposing organic matter (Delgado‐Baquerizo et al. 2018; Oliverio et al. 2020).

Beyond bacterial and fungal decomposers, microbial predators such as nematodes and protists might also influence necromass decomposition (Kennedy and Maillard 2023). In terrestrial ecosystems, microbial predators such as nematodes and protists have been shown to affect decomposition rates by preying on microbial decomposers like bacteria and fungi (Ekelund and Rønn 1994; Kuikman et al. 1990). Controlled studies, in which microbial decomposers are inoculated either alone or alongside one or more microbial predators, have demonstrated that predation modulates the extent of plant litter decomposition (Bouwman and Zwart 1994; Geisen et al. 2021; Kuikman et al. 1990; Wang et al. 2024). These findings are further supported by community‐level studies showing statistical associations between microbial predator communities, plant litter decomposition rates, and SOC stocks (Jiang et al. 2018; Liao et al. 2023; Mielke et al. 2022). Moreover, microbial predators can exert indirect effects on decomposers, resulting in contrasting impacts on organic matter decay—sometimes increasing (Bouwman and Zwart 1994; Geisen et al. 2021) and at other times decreasing decomposition (Wang et al. 2024). These opposing effects arise because predators can alter decomposer activity either by reducing their abundance or reshaping community composition. Additionally, direct interactions between predators and prey—such as chemical warfare—can divert microbial energy away from decomposition and toward defence pathways (Jousset et al. 2010; Mazzola et al. 2009). Modelling studies also suggest that microbial predation influences decomposition by accelerating nutrient mineralisation, including nitrogen, which is often a limiting resource for microbial decomposers (Wang et al. 2009). However, because decomposer communities in fungal necromass differ from those in bulk soil in bacterial and fungal abundance, diversity, and taxonomic composition, the predator communities they support, which are shaped by feeding strategies (bacterivory vs. fungivory) and prey specificity (Fujino et al. 2024), may also differ. Yet how these predator differences may influence necromass decomposition remains unexplored, despite important implications for microscale brown food‐web dynamics and SOC formation.

In this study, we aimed to determine the differences in microbial community structure—abundance, richness, and taxonomic and functional composition—between soil and fungal necromass, focusing on bacteria, fungi, and their predators (protists and nematodes). We also sought to test whether predator communities indirectly influence necromass decomposition rates by modulating secondary decomposer abundances in situ. To address these objectives, we deployed fungal necromass of contrasting quality—low melanin content (high quality) and high melanin content (low quality)—across 12 plots in a forested site in Minnesota, USA. Previous work has shown that melanin content governs necromass decay rates and shapes decomposer communities, but its effects on higher trophic levels remain unexamined (Fernandez and Koide 2014; Fernandez and Kennedy 2018; Fernandez et al. 2019; Maillard, Pflender, et al. 2022; Maillard, Michaud, et al. 2023). Using a phenotypically variable mycorrhizal fungus (see Materials and Methods), our approach enabled us not only to assess how necromass quality directly shapes secondary decomposer community composition, but also to examine how these substrate differences influence the assembly of microbial predator communities. We harvested necromass after 4 and 12 weeks of decomposition to capture both the early and late decay stages (See et al. 2021), along with surrounding soils. Microbial decomposer abundance (bacteria and fungi) was quantified in necromass and soil using quantitative PCR (qPCR), and microbial decomposer and predator community structure was characterised using metabarcoding of multiple microbial markers. Finally, we employed structural equation modelling to test whether predators indirectly affect necromass mass loss by altering decomposer abundances.

The rapidly growing literature of fungal necromass decomposition indicates that fungal necromass harbours greater bacterial abundances, reduced microbial decomposer diversity, and different microbial decomposer assemblages relative to bulk soil, but also that these patterns vary with necromass quality and decay stage. On this basis, we tested three hypotheses:

*Predator communities (protists and nematodes) that assemble on fungal necromass will be taxonomically and functionally distinct from those in neighbouring bulk soil, with a higher relative abundance of bacterivorous taxa and reduced diversity due to new assemblage formation relative to soil*.

*Necromass biochemical quality—determined by melanin content—and decomposition time will influence microbial predator composition respectively through prey preferences and colonisation rates, with nematodes exhibiting greater migration potential than protists from the surrounding bulk soil and thus greater diversity in the early stages of decomposition*.

*Predator community structure will covary with bacterial and fungal abundances and, through those links, indirectly with necromass mass loss rates, hinting at a potential regulation of fungal necromass decomposition by microbial predators*.

## 2 Materials and Methods

### 2.1 Fungal Necromass Production

We generated fungal necromass from *Hyaloscypha bicolor* (formerly *Meliniomyces bicolor*), a widely distributed soil fungus forming mutualistic interactions with various trees and shrubs (Grelet et al. 2009). Following the protocol of Fernandez and Kennedy (2018), we produced two distinct necromass types differing in melanin content (i.e., low vs. high). Briefly, to manipulate melanin levels, we cultured *H. bicolor* mycelia for 30 days in 125 mL flasks containing half‐strength potato dextrose broth (PDB, Difco) adjusted to pH 5, under two different submersion volumes: 40 mL for lowly melanised biomass and 120 mL for highly melanised biomass. Cultures were incubated at room temperature on orbital shakers at 150 RPM. We then harvested the mycelia, rinsed them thoroughly with autoclaved deionised water, and freeze‐dried. According to Fernandez and Kennedy (2018), the low melanin necromass contained 52.6% ± 1.85% C, 4.8% ± 0.07% N (C:N ratio = 11.0 ± 0.25), and 71 ± 39.75 mg melanin g^−1^, while the high melanin necromass contained 51.3% ± 0.22% C, 3.8% ± 0.03% N (C:N ratio = 13.7 ± 0.13), and 229 ± 7.79 mg melanin g^−1^. These differences were visually confirmed, with the low melanin necromass exhibiting a light grey colour and the high melanin necromass displaying a black coloration. Each necromass type (low vs. high melanin) was portioned into ~100 mg dry matter aliquots and sealed in polyester mesh bags (hereafter ‘mycobags’) measuring 5 × 10 cm, with 53‐μm pore size (R510 Forage Bag, ANKOM Technology, Macedon, NY, USA). The 53‐μm mesh effectively excluded fine roots and soil particles, thereby facilitating measurements of necromass decomposition rates (Beidler et al. 2020).

### 2.2 Fungal Necromass Decomposition

We conducted the decomposition experiment in a mixed‐age white pine ( *Pinus strobus* ) forest at the Cedar Creek Ecosystem Science Reserve (Cedar Creek; 45°25′ N, 93°10′ W) in eastern Minnesota, USA. Cedar Creek is a 2300‐ha reserve and National Science Foundation Long‐Term Ecological Research site located on the Anoka Sand Plain, which is characterised by excessively drained soils containing up to 90% sand. In early August 2020, we buried mycobags containing either low or high melanin necromass within the top 5 cm of mineral soil in 12 plots, placing two mycobags of each necromass type per plot (48 bags in total). We retrieved them after one and 3 months (i.e., 4 and 12 weeks) on September 3 and November 5, 2020, respectively. These decomposition times were chosen based on previous necromass experiments, including at Cedar Creek (Fernandez and Kennedy 2018), to capture the earlier and later stages of decomposition (Cantoran et al. 2023). Each retrieved bag was individually packaged, stored at 4°C, and promptly transported to the lab. One bag was lost during the course of the experiment and thus excluded from all analyses.

At each necromass sampling time, we also collected three soil cores (0–5 cm) from each plot, carefully removing any organic horizon. The cores were taken at least 50 cm from the buried mycobags to avoid local necromass effects. We pooled the three cores per plot into a single composite sample in situ (*n* = 24 total per sampling time), stored them at 4°C, and processed them within days of harvest. In the laboratory, necromass from each mycobag was carefully extracted with sterile pipette tips, transferred to sterilised 2 mL tubes, and stored at −20°C before freeze‐drying. Soil samples were passed through a 2 mm mesh sieve, placed in 15 mL centrifuge tubes, and stored at −20°C prior to freeze‐drying.

### 2.3 Bacterial and Fungal Abundances

Total genomic DNA was extracted from freeze‐dried soil and fungal necromass using PowerSoil Pro kits (Qiagen, Hilden, Germany), following the manufacturer's protocol. Bacterial and fungal abundances were then quantified via qPCR on a StepOne Real‐Time PCR system (Thermo Fisher Scientific, Waltham, MA, USA), targeting bacterial 16S rRNA genes with the 968F/1401R primer set (Heuer et al. 1999) and fungal 18S rRNA genes with the FR1/FF390 primer set (Chemidlin Prévost‐Bouré et al. 2011). Each 20 μL qPCR reaction contained 1 μL of a 1:10 dilution of template DNA, standard bacterial or fungal plasmid DNA (10^9^ to 10^3^ gene copies μL^−1^) or molecular‐grade water as a negative control, and iQ SYBR Green Supermix (Bio‐Rad, Hercules, CA, USA). The amplification programme included 5 min at 95°C, followed by 40 cycles of 20 s at 95°C, 30 s at the primer‐specific annealing temperature (56°C for 16S; 50°C for 18S), and 60 s at 72°C. Primer specificity was assessed via a melting‐curve analysis from 70°C to 95°C at increments of 0.3°C s^−1^. All samples were analysed in technical duplicates, and results were averaged to obtain copy numbers (bacterial 16S or fungal 18S) per gram of dry soil or necromass. Fungal metabarcoding results (see below) were checked for residual necromass DNA, confirming it had degraded before the first sampling time (4 weeks), ensuring no bias in fungal qPCR results and revealing no *Hyaloscypha bicolor* DNA in decomposing necromass samples.

### 2.4 Microbial Community Analyses

We performed high‐throughput sequencing (HTS) of bacterial, fungal, and microbial eukaryotic taxonomic markers to characterise community composition in both soil and fungal necromass. In total, we analysed 47 necromass samples and 24 soil samples. DNA extraction and PCR blanks (molecular‐grade water) were included as controls. To capture bacterial diversity, we amplified the V4 region of the 16S rRNA gene using the 515F‐806R primers (Caporaso et al. 2012). Fungal communities were assessed via the ITS2 region using the 5.8S‐Fun and ITS4‐Fun primers (Taylor et al. 2016). For microbial eukaryotes (protists and nematodes), we targeted the 18S rRNA gene with the 616*f‐1132r primer pair (Hugerth et al. 2014), found to amplify the widest taxonomic range of eukaryotes (Vaulot et al. 2022). First‐round PCRs (20 μL) contained 10 μL Phusion Hot Start II High‐Fidelity PCR Master Mix (Thermo Scientific, Waltham, MA, USA), 0.5 μL of each 20 µM primer, 1 μL template DNA, and 8 μL PCR‐grade water. The thermocycling steps were: (1) 98°C for 30 s; (2) 98°C for 30 s; (3) 50°C (16S), 55°C (ITS), or 52°C (18S) for 30 s; (4) 72°C for 30 s; then repeating steps 2–4 for 34 additional cycles, with a final extension at 72°C for 10 min. When initial PCRs yielded no detectable amplicons, the template DNA was diluted and reamplified. Amplicons from successful reactions underwent a second PCR (same conditions) to add unique Golay barcodes and sequencing adapters. We purified and normalised PCR products using the Charm Just‐a‐Plate Purification and Normalisation Kit (Charm Biotech, San Diego, CA, USA) and pooled them at equimolar concentrations. Sequencing was performed using Illumina MiSeq 2 × 250 bp V2 chemistry at the University of Minnesota Genomics Center.

Raw reads were processed using the AMPtk v1.4.2 pipeline (Palmer et al. 2018). Based on quality assessments of a fungal mock community, we retained only the forward (R1) reads for bacteria, fungi, protists, and nematodes to avoid data loss caused by poor R2 quality; previous studies have shown that using R1 alone—both in mock communities and in environmental samples—yields similar or even better recovery of microbial diversity than merging paired reads (Nguyen et al. 2015; Pauvert et al. 2019). After primer removal and trimming to 250 bp, sequences were denoised via DADA2 (Callahan et al. 2016) and then clustered into OTUs at 97% similarity (Schloss 2021). We removed OTUs with abundances below 0.5% (to control for barcode index bleed; Palmer et al. 2018). Taxonomic assignments utilised a hybrid approach combining USEARCH global alignment (RDP for bacteria, UNITE for fungi, and PR2 for microbial eukaryotes) with UTAX and SINTAX classifiers. For the bacterial dataset, we excluded OTUs identified as non‐bacterial, chloroplast, or mitochondrial. For the fungal dataset, we removed OTUs without fungal annotations. Protist and nematode OTUs were separated from the 18S dataset. OTUs detected as contaminants in extraction/PCR blanks were summed and subtracted from environmental samples. Bacterial and fungal datasets were rarefied to 6149 and 1159 reads per sample, respectively. Because of a large proportion of contaminating 18S bacterial/fungal reads and the subsequent division of 18S data into protist vs. nematode OTU tables, protist and nematode reads were instead converted to relative abundances. Samples containing fewer than 1000 fungal reads or fewer than 100 protist or nematode reads were removed. Final sample numbers are presented in Table S1. OTU accumulation curves plateaued for all microbial groups and treatments (Figure S1), and Good's coverage values remained high (0.982–0.996 for bacteria and fungi, 0.994–1.000 for nematodes, and 0.914 ± 0.014) in bulk soil, rising to ~0.99 in necromass protist libraries (Table S2), indicating that sequencing depths were adequate.

### 2.5 Functional Group Assignments

To explore functional traits within microbial communities, we assigned bacterial OTUs to copiotrophic or oligotrophic lifestyles based on Li et al. (2021). Briefly, OTUs in the phyla Bacteroidetes, Firmicutes, Gemmatimonadetes, and classes α‐, β‐, and γ‐Proteobacteria were considered copiotrophic, whereas those in phyla Acidobacteria, Planctomycetes, Chloroflexi, and class δ‐Proteobacteria were considered oligotrophic. Fungal trophic modes were classified using FUNGuild (Nguyen et al. 2015) and the FungalTraits database (Põlme et al. 2020) into saprotrophic, ectomycorrhizal, mycoparasitic, or animal‐parasitic fungi. Protists were assigned to four major functional groups—primary consumers (bacterivores or small‐yeast feeders), secondary consumers (predators of larger microbes), parasites (infecting diverse hosts), and phototrophs—based on Adl et al. (2018), Dumack et al. (2020), Hiltunen et al. (2012), Geisen et al. (2018), Nguyen et al. (2020), and Oliverio et al. (2020). Nematodes were grouped into five functional categories (bacterivores, fungivores, plant feeders, omnivores and predators) according to Yeates et al. (1993) and van den Hoogen et al. (2020).

### 2.6 Data Analyses

All statistical analyses and data visualisations were carried out in R (R Core Team 2020) with a significance threshold of *α* = 0.05. Because ‘sampling time’ captured different processes, that is, seasonal variation in soil, but a mix of decomposition interval and seasonal effects for necromass, we first tested its influence on the soil data. Microbial abundances and OTU richness were compared with paired *t*‐tests, and community composition with PERMANOVA (Bray–Curtis dissimilarity, vegan package; Oksanen et al. 2013). Except for bacterial OTU composition, none of these metrics differed significantly across sampling times (Table S3). We therefore omitted sampling time as a factor in subsequent soil analyses to prevent confounding with the decomposition‐time treatment used for necromass.

For univariate analyses (e.g., microbial abundance, OTU richness, functional group abundance), we used a nested factorial design with ‘habitat’ (soil vs. fungal necromass) as the main factor and a two‐way factorial combination of'decomposition ‘time’ (4 vs. 12 weeks) and ‘necromass type’ (low vs. high melanin) nested within fungal necromass. Linear mixed‐effects models (lme4; Bates et al. 2015) included ‘plot’ as a random intercept to account for among‐plot variability. Model assumptions were checked visually with residual and *Q*–*Q* plots and with Levene's test (car; Fox et al. 2012) for homoscedasticity. When assumptions were violated, response variables were log‐transformed. We assessed fixed effects via Type III ANOVA with Satterthwaite's method (lmerTest package, Kuznetsova et al. 2017). Pairwise treatment differences were assessed by Tukey‐adjusted comparisons of estimated marginal means (emmeans package, Lenth 2021), and compact letter groupings (multcompView package, Graves et al. 2015) were employed to summarise these pairwise comparisons.

Microbial community composition was analysed with PERMANOVA on Bray–Curtis dissimilarities. We first compared habitats (soil vs. necromass), and then—within the necromass dataset—tested the effects of decomposition time, necromass quality, and their interaction. The OTU compositions of bacterial, fungal, protist, and nematode communities were subsequently visualised with non‐metric multidimensional scaling (NMDS). We applied indicator species analysis (from the indicspecies package, De Caceres et al. 2016) to identify specific genera that were significantly associated with each habitat (soil or fungal necromass), necromass decomposition time (4 vs. 12 weeks), and melanin content (low vs. high).

We used structural equation modelling (SEM; lavaan package—Rosseel 2012) to investigate the potential indirect effect of microbial predators (protists and nematodes) on fungal necromass loss via their predation on decomposer communities. Our a priori conceptual model—which posited a top‐down control of microbial decomposers by predators and a cascading effect on necromass loss (Figure S2)—included the following: (i) direct paths from bacterial and fungal abundance (qPCR‐derived) to necromass mass loss (hypothesising greater microbial abundance leads to higher mass loss); (ii) a co‐variable link between bacterial and fungal abundance to capture non‐directional interactions (e.g., competition or facilitation); (iii) paths from protist and nematode community composition (first or second axis from NMDS) to bacterial and fungal abundances to highlight predatory activities; (iv) a co‐variable link between protist and nematode communities; (v) indirect effects of protist and nematode communities on necromass loss through their influence on bacterial or fungal abundances. We used maximum likelihood estimation with robust standard errors to address non‐normality, and tested separate models for each necromass type × decomposition time. Models with *χ* ^2^ > 0.05 and RMSEA ≤ 0.08 were deemed acceptable; non‐significant paths that improved model fit were retained, while non‐significant paths that did not improve fit were removed.

## 3 Results

### 3.1 Necromass Mass Loss

Fungal necromass mass remaining was significantly influenced by decomposition time (*F* = 5.7; *p* < 0.05) and necromass type (*F* = 63.4; *p* < 0.001). Most mass loss occurred during the first 4 weeks of incubation, with high melanin necromass retaining more mass than low melanin necromass at both sampling times (Figure S3).

### 3.2 Microbial Abundances in Soil and Fungal Necromass

**FIGURE 1:**
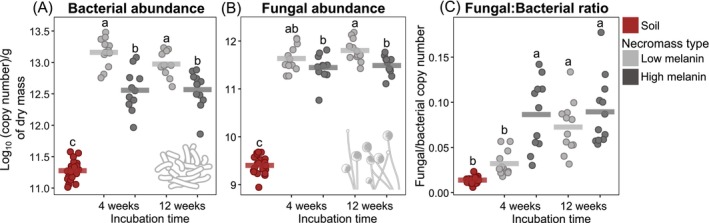
(A) Bacterial and (B) fungal abundances (log_10_[copy numbers] quantified by qPCR) in fungal necromass with low or high melanin content, after 4 or 12 weeks of incubation, compared with bulk soil, and (C) the ratio of fungal to bacterial abundances for the same treatments. Each jittered point represents an individual sample, and the horizontal bar indicates the mean. All data were analysed using linear mixed‐effects models with ‘Plot’ as a random factor, followed by ANOVA and Tukey's HSD. Different letters indicate significant differences at *p* < 0.05.

Bacterial (*F* = 253.7; *p* < 0.001) and fungal (*F* = 674.8; *p* < 0.001) abundances, quantified by qPCR, were consistently and significantly higher in necromass relative to soil, showing 20‐ to 80‐fold increases for both domains (Figure 1A,B). Within necromass, bacterial (*F* = 56.9; *p* < 0.001) and fungal (*F* = 14.5; *p* < 0.001) abundances were significantly higher in low melanin necromass compared to high melanin necromass, while decomposition time had only limited effects. The ratio of fungal‐to‐bacterial abundances was generally higher in necromass than in soil (*F* = 75.2; *p* < 0.001), except for low melanin necromass after 4 weeks of incubation (Figure 1C). High melanin necromass exhibited a significantly higher fungal‐to‐bacterial ratio than low melanin necromass (*F* = 23.7; *p* < 0.001) (Figure 1C).

### 3.3 Microbial Community Diversity and Composition in Soil and Necromass

**FIGURE 2:**
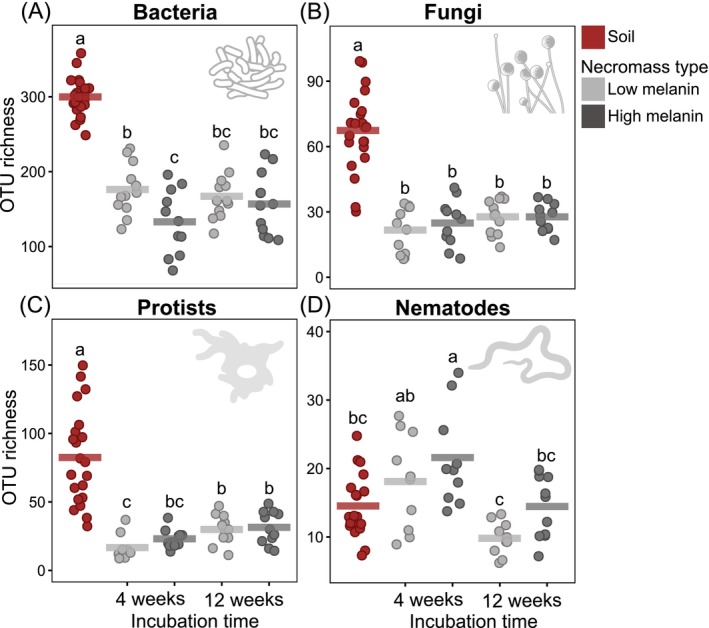
Operational taxonomic unit (OTU) richness in fungal necromass with low or high melanin content, after 4 or 12 weeks of incubation, compared with bulk soil: (A) bacteria, (B) fungi, (C) protists, and (D) nematodes. Each jittered point represents an individual sample, and the horizontal bar indicates the mean. OTU richness was analysed using linear mixed‐effects models with ‘Plot’ as a random factor, followed by ANOVA and Tukey's HSD. Different letters indicate significant differences at *p* < 0.05.

OTU richness for bacteria (*F* = 160.8; *p* < 0.001), fungi (*F* = 81.4; *p* < 0.001), and protists (*F* = 42.4; *p* < 0.001) was significantly lower in necromass compared to soil, decreasing by about 50% for bacteria and fungi and by 65% for protists (Figure 2A–C). These habitat‐driven differences were consistent across necromass decomposition times and melanin levels. In contrast, nematode OTU richness did not show a clear habitat effect (*F* = 0.002; *p* = 0.97) and was significantly higher in high melanin necromass at 4 weeks compared to soil (Figure 2D). Necromass type and decomposition time further modulated OTU richness across microbial groups. Within bacterial communities, OTU richness was significantly higher in low melanin necromass compared to high melanin necromass (*F* = 8.2; *p* < 0.01) (Figure 2A). Protist OTU richness was strongly influenced by decomposition time (*F* = 13.6; *p* < 0.001), with higher richness in necromass after 12 weeks of decomposition than after 4 weeks (Figure 2C). Nematode OTU richness was significantly affected by both necromass type (*F* = 6.1; *p* < 0.05) and decomposition time (*F* = 21.9; *p* < 0.001), with higher values in high melanin necromass than low melanin necromass and lower values after 12 weeks relative to 4 weeks of decomposition (Figure 2D). Fungal OTU richness was not significantly affected by necromass melanin content (*F* = 0.17; *p* = 0.68) nor decomposition time (*F* = 1.60; *p* = 0.21) (Figure 2B).

**FIGURE 3:**
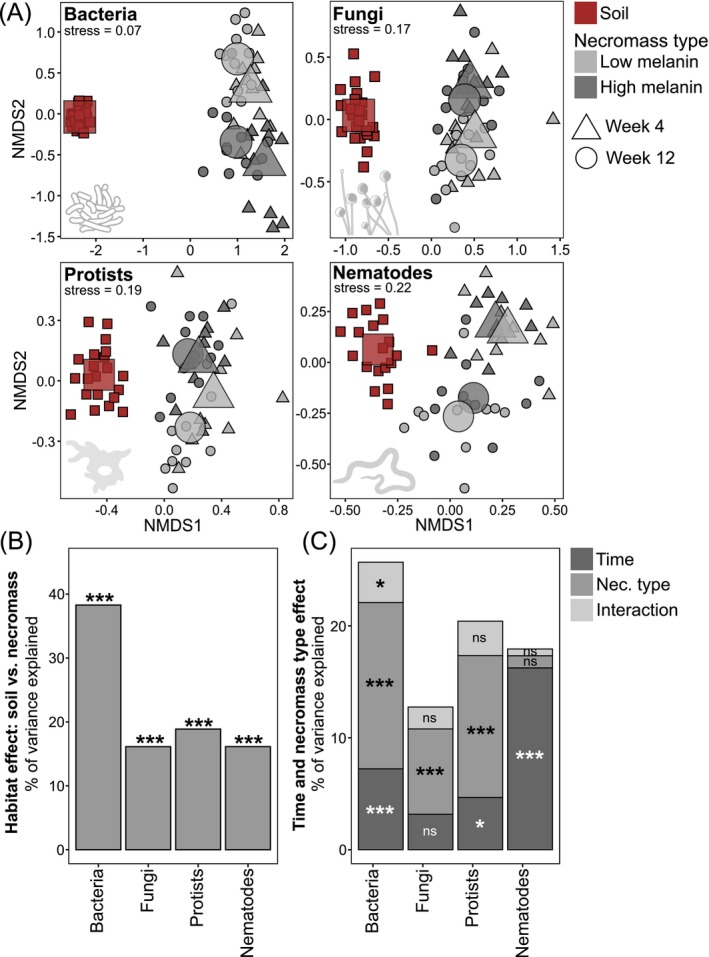
(A) Non‐metric multidimensional scaling (NMDS) ordination of bacterial, fungal, protist, and nematode community composition in fungal necromass with low or high melanin content, after 4 or 12 weeks of incubation, together with bulk soil. Small points represent individual samples, and larger symbols indicate group centroids. (B) Variance in community composition explained by habitat (soil or necromass), determined by PERMANOVA. (C) Variance in community composition explained by incubation time and melanin content in necromass, also determined by PERMANOVA. *p < 0.05, ***p < 0.001.

NMDS in combination with PERMANOVA revealed significant differences between soil and necromass microbial communities across all four microbial groups (*p* < 0.001), with habitat (soil vs. necromass) explaining ~17% of variation in fungi, protist, and nematode OTU composition and up to ~38% in bacterial OTU composition (Figure 3A,B). Within necromass, bacterial and protist OTU composition was both significantly shaped by decomposition time and melanin content, whereas fungal communities were only significantly affected by melanin content, and nematode communities only by decomposition time (Figure 3C).

**FIGURE 4:**
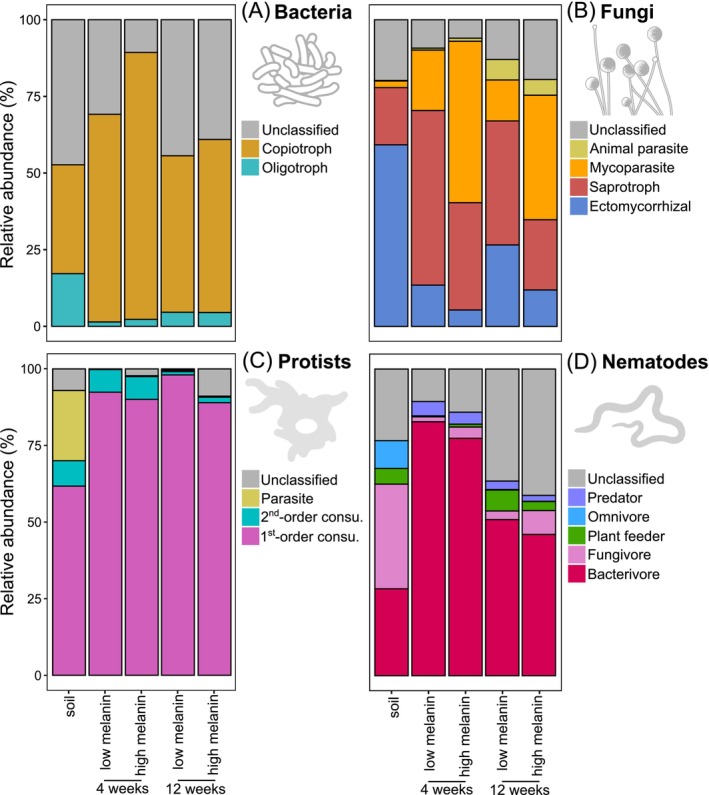
Mean relative abundances of the bacterial, fungal, protist, and nematode functional groups in fungal necromass with low or high melanin content, after 4 or 12 weeks of incubation, compared with bulk soil: (A) bacteria, (B) fungi, (C) protists, and (D) nematodes. The number of biological replicates for each treatment is listed in Table S1.

**FIGURE 5:**
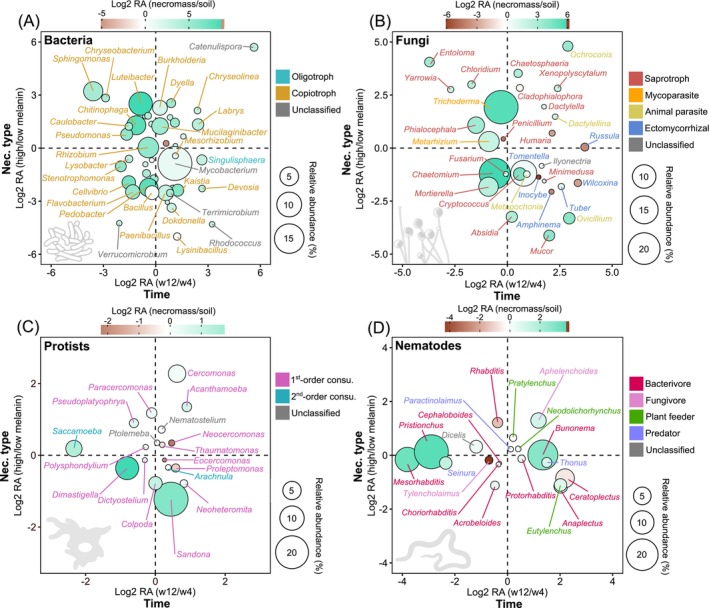
Differential analysis of bacterial, fungal, protist, and nematode genera in fungal necromass based on the log_2_(fold change in relative abundance) for high versus low melanin content and 12 versus 4 weeks of incubation: (A) bacteria, (B) fungi, (C) protists, and (D) nematodes. Only genera with an average relative abundance (RA) of at least 0.1% across all necromass samples are shown. The size of each bubble corresponds to the average relative abundance of the genus, while the bubble colour indicates whether the genus is more abundant in soil (brown) or necromass (blue). Genus names are colour‐coded by their functional groups. See Table S4 for genera significantly affected by habitat (bulk soil vs. necromass), Table S5 for necromass quality (low vs. high melanin), and Table S6 for incubation time (4 vs. 12 weeks), based on Indicspecies analysis.

Bacterial communities in both soil and necromass were primarily dominated by Actinobacteria, α‐ and β‐proteobacteria, and Sphingobacteriia (Figure S4A). In terms of functional groups, necromass was significantly enriched in copiotrophs (*F* = 30.2; *p* < 0.001) and depleted in oligotrophs (*F* = 58.3; *p* < 0.001) (Figure 4A) relative to soil. For necromass samples, decomposition time also significantly affected the relative abundances of both oligotrophic (*F* = 21.1; *p* < 0.001) and copiotrophic (*F* = 21.1; *p* < 0.001) bacteria, but in opposite directions: the former increased with decomposition time, while the latter decreased. Many bacterial genera (e.g., *Chitinophaga*, *Cellvibrio*, *Dyella*, *Luteibacter*, *Rhizobium*) were significantly associated with necromass relative to soil (Figure 5A, Table S4). Specific associations with necromass type were also observed: *Bacillus*, *Devosia*, *Dokdonella*, *Cellvibrio*, *Flavobacterium*, *Kaistia*, *Lysinibacillus*, and *Rhodococcus* were significantly enriched in low melanin necromass, whereas *Burkholderia*, *Dyella*, *Catenulispora*, and *Luteibacter* were enriched in high melanin necromass (Figure 5A, Table S5). Further, decomposition time also influenced genus associations: *Stenotrophomonas* was associated with 4‐week necromass, while *Catenulispora*, *Devosia*, and *Singulisphaera* were associated with necromass decomposed for 12 weeks (Figure 5A, Table S6).

Fungal communities were dominated by Agaricomycetes in soil, whereas necromass was mostly composed of Sordariomycetes, Agaricomycetes, and Mortierellomycetes (Figure S4B). Soil fungal assemblages contained significantly more ectomycorrhizal fungi (*F* = 26.7; *p* < 0.001), while necromass was significantly enriched in mycoparasitic taxa (*F* = 60.6; *p* < 0.001) (Figure 4B). Within necromass, high melanin samples contained higher proportions of mycoparasites (*F* = 23.9; *p* < 0.001), whereas low melanin samples had higher proportions of ectomycorrhizal and saprotrophic fungi (*F* = 5.9; *p* < 0.05). Animal parasitic fungi became more abundant in necromass after 12 weeks of decomposition compared with 4 weeks (*F* = 6.2; *p* < 0.05). Dominant fungal genera in necromass included *Trichoderma* and *Chaetomium*, both significantly enriched relative to soil (Figure 5B, Table S4). Specific associations with necromass type were also observed: *Absidia*, *Fusarium*, and *Ovicillium* were enriched in low melanin necromass, while *Chloridium*, *Cladophialophora*, and *Dactylella* were enriched in high melanin necromass (Figure 5B, Table S5). Additionally, decomposition time influenced genus relative abundance, with *Ilyonectria* and *Russula* significantly more abundant in necromass decomposed for 12 weeks (Figure 5B, Table S6).

Protist communities in soil were dominated by Cercozoa, Lobosa, and Apicomplexa, whereas in necromass Cercozoa, Lobosa, and Discoba were the most abundant phyla (Figure S4C). In terms of functional groups, first‐order consumer protists were the most abundant in both soil and necromass. First‐order consumer protists (*F* = 22.3; *p* < 0.001) were significantly more abundant in necromass, while parasites (*F* = 116; *p* < 0.001) were less abundant relative to soil (Figure 4C). At the genus level, several protist taxa were significantly enriched in necromass compared with soil, including *Sandona*, *Dimastigella*, *Saccamoeba*, *Colpoda*, *Cercomonas*, *Pseudoplatyophrya*, and *Acanthamoeba* (Figure 5C, Table S4). High melanin necromass was specifically enriched in *Acanthamoeba*, *Cercomonas*, and *Ptolemeba* relative to low melanin necromass (Figure 5C, Table S5). The genus *Saccamoeba* was significantly more abundant in necromass decomposed for 4 weeks, and *Arachnula* and *Neocercomonas* were more abundant in necromass decomposed for 12 weeks (Figure 5C, Table S6).

Nematode communities were dominated by Enoplea and Chromadorea in soil and Chromadorea in necromass (Figure S4D). Fungivores were the most abundant nematodes in soils, whereas they were significantly less abundant in necromass (*F* = 37.1; *p* < 0.001) (Figure 4D). Bacterivore nematodes were the most abundant in necromass, and necromass after 4 weeks of decomposition had a significantly higher proportion of bacterivores than did necromass at 12 weeks (*F* = 9.7; *p* < 0.01). Several nematode genera, including *Acrobeloides*, *Bunonema*, *Mesorhabditis*, *Pristionchus*, *Thonus*, *Protorhabditis*, and *Paractinolaimus*, were significantly enriched in necromass compared to soil (Figure 5D, Table S4). The genus *Pratylenchus* was specifically associated with high melanin necromass (Figure 5D, Table S5). Decomposition time also influenced the abundance, with *Dicelis*, *Mesorhabditis*, *Pristionchus*, *Seinura*, and *Tylencholaimus* being significantly more abundant in necromass decomposed for 4 weeks relative to 12 weeks (Figure 5D, Table S6).

### 3.4 Structural Equation Modelling

**FIGURE 6:**
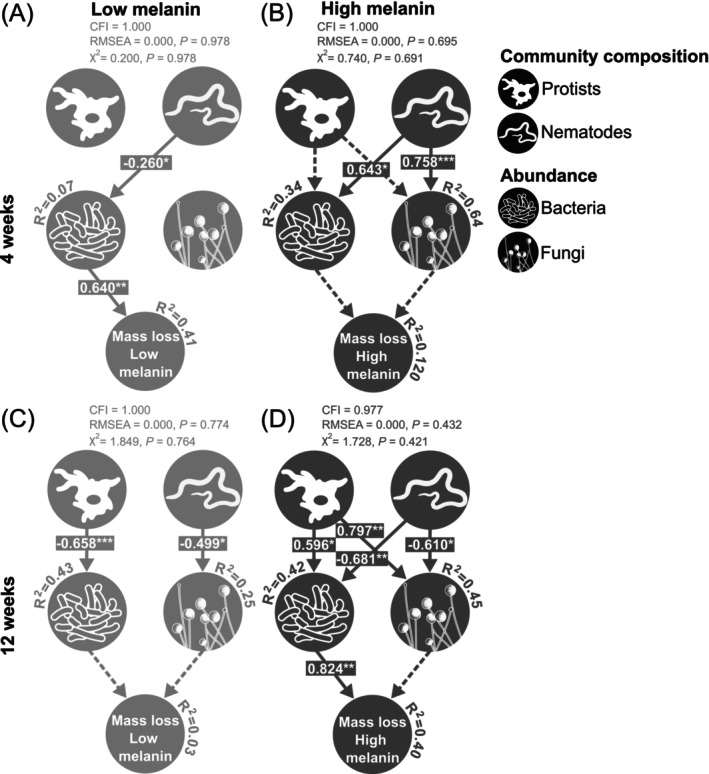
Structural equation model (SEM) depicting the effects of protist and nematode communities on bacterial and fungal abundances, and the subsequent effects of bacterial and fungal abundances on fungal necromass mass loss. The model includes necromass with low or high melanin content, incubated for 4 or 12 weeks (panels: A = low melanin, 4 weeks; B = high melanin, 4 weeks; C = low melanin, 12 weeks; D = high melanin, 12 weeks). Standardised path coefficients are shown on each arrow. Indirect effects of protist and nematode community composition on necromass mass losses, mediated by modifications in bacterial or fungal abundances, are directly reported in the text. Non‐significant paths retained to improve model fit are shown as dotted arrows, while non‐significant paths that did not improve fit were removed. The *r* ^2^ values indicate the proportion of variance explained. *p < 0.05, **p < 0.01, ***p < 0.001.

A series of separate SEMs (one for each necromass type [low vs. high melanin] and decomposition time [4 vs. 12 weeks]) included paths from protist and nematode community structures to bacterial and fungal abundances, and from those abundances to necromass mass loss (Figure 6), with all models meeting convergence criteria (*χ* ^2^ > 0.05; RMSEA < 0.08). After 4 weeks (Figure 6A,B), nematode community structure (but not protist structure) was associated with microbial abundances, showing a negative association with bacterial abundance on low melanin necromass and positive associations with both bacterial and fungal abundances on high melanin necromass. By 12 weeks (Figure 6C,D), both nematode and protist community compositions were associated with decomposer abundances. On low melanin necromass, protist composition was negatively associated with bacterial abundance while nematode composition was negatively associated with fungal abundance. On high melanin necromass, protist composition was positively associated and nematode composition negatively associated with both bacterial and fungal abundances. Except for the low melanin 4‐week model, predator community metrics accounted for 25%–64% of the variance in microbial abundances. Bacterial abundance was positively associated with necromass mass loss in low melanin necromass at 4 weeks (explaining 41% of its variance) and in high melanin necromass at 12 weeks (40%), with marginally supported indirect associations via nematodes (*p* = 0.089) at 4 weeks and via both nematodes (*p* = 0.064) and protists (*p* = 0.097) at 12 weeks.

## 4 Discussion

To our knowledge, this study is the first to integrate microbial predators into fungal necromass decomposition and to assess their relationships with decomposer abundances and decay trajectories. Our results reveal distinct, temporally dynamic shifts in necromass breakdown and its associated decomposer and predator microbial communities. Furthermore, on both necromass types, we demonstrate significant statistical associations between predator community composition and the abundances of bacteria and fungi involved in necromass decay, highlighting the potential role of predators in shaping fungal necromass turnover.

### 4.1 Microbial Decomposer Abundance During Fungal Necromass Decay

In line with previous studies, we observed an initial phase of rapid mycelial residue mass loss followed by a plateau, underscoring the asymptotic nature of fungal necromass decomposition (Brabcová et al. 2016; Maillard et al. 2021; Ryan et al. 2020; See et al. 2021). Consistent with the recalcitrant properties of melanin (Fernandez and Kennedy 2018; Ryan et al. 2020), our data show substantially higher mass retention in high melanin necromass, further supporting the idea that melanin slows decay rates and increases the fraction of necromass stabilising in the particulate fraction of SOM (Fernandez and Kennedy 2018; Maillard, Michaud, et al. 2023). Although some studies have proposed that bacterial decomposers dominate fungal necromass decay (Brabcová et al. 2016), we found strong enrichment in decomposing necromass for both bacterial and fungal decomposers relative to surrounding soil. This finding aligns with Maillard, Michaud, et al. (2023), who used quantitative stable isotope probing (qSIP) to show that both bacterial and fungal communities derive carbon and nitrogen from fungal necromass in situ. We also observed that bacterial and fungal abundances remained high at 12 weeks, suggesting that necromass continues to support secondary decomposers capable of exploiting relatively recalcitrant substrates as well as likely targeting new necromass originating from the colonising microbes themselves. Melanin content also drove differences in microbial decomposer abundance, with low melanin necromass supporting higher overall microbial loads, reflecting its relatively greater nutritional value or reduced biochemical protection (Maillard, Michaud, et al. 2023). In contrast, high melanin necromass supported fewer bacteria and fungi initially but maintained a higher fungal‐to‐bacterial ratio, possibly indicating greater fungal capacity for decomposing melanised necromass. Taken together, these observations confirm that fungal necromass forms a discrete ‘hotspot’ of microbial activity in forest soils and illustrate how melanin content and decomposition stage modulate both bacterial and fungal abundance. We speculate that discrepancies with earlier work (e.g., Brabcová et al. 2016) may stem from differences in substrate type (fruiting bodies vs. mycelial necromass here) and initial necromass amounts (3 vs. 0.1 g here), highlighting the need for further comparative studies to clarify how fungal necromass traits influence bacterial and fungal dominance in necromass decay processes.

### 4.2 Microbial Decomposer Community Dynamics During Fungal Necromass Decay

In addition to abundance patterns, our results corroborate previous findings that the composition and diversity of bacterial and fungal decomposers in necromass differ markedly from those in bulk soil (Beidler et al. 2020; Brabcová et al. 2016). Specifically, both bacterial and fungal OTU richness were reduced by > 50% in necromass compared to soil, with no strong effects of necromass type or decomposition time. This finding is consistent with the idea that necromass decomposition is carried out by a more specialised subset of the soil microbiome (Beidler et al. 2020; Cantoran et al. 2023; Maillard, Colin, et al. 2023). It is, however, important to note here that because these estimates are based on environmental metabarcoding, the higher OTU richness observed in soils may also reflect contributions from relic DNA (Carini et al. 2016). The necromass decomposer community was enriched in copiotrophic genera specialised in fungal necromass decomposition (e.g., *Chitinophaga*, *Dyella*, *Burkholderia*, *Luteibacter*) and the fungal community included saprotrophic, mycoparasitic, and ectomycorrhizal taxa (*Fusarium*, *Trichoderma*, *Mucor*, *Mortierella*, *Tomentella*), many of which are widely recognised as key members of a fungal necrobiome (Cantoran et al. 2023; Fernandez and Kennedy 2018). Furthermore, melanin content emerged as a major factor structuring these decomposer communities, aligning with prior research showing that melanisation can select for microbial taxa capable of degrading complex aromatic cell‐wall components (Fernandez et al. 2019; Maillard, Michaud, et al. 2023; Ryan et al. 2020). By contrast, decomposition time had a stronger effect on bacterial community composition than on fungal composition, possibly indicating that necromass‐associated bacterial communities are more sensitive to the progressive changes in necromass biochemical composition typically observed during decomposition, as well as to the accompanying shifts in resource availability (Maillard, Michaud, et al. 2023; Ryan et al., 2020). Collectively, these results support the emerging research frameworks on fungal necromass decomposition, emphasising that substrate quality, whether modulated by initial biochemical differences in senescing mycelial necromass or by biochemical differences induced by decomposition processes, plays a dominant role in shaping microbial decomposer successional trajectories.

### 4.3 Nematode Community Dynamics During Fungal Necromass Decay

Because this study is the first to examine nematode communities in fungal necromass, we compare the observed patterns to those in plant‐litter decomposition, which similarly involves localised, carbon‐ and nutrient‐rich patches of organic matter adjacent to a more oligotrophic soil matrix. The first striking pattern was that, unlike other microbial groups, nematode OTU richness was higher in necromass during early decay stages compared to soil, in disagreement with our first hypothesis (H1). This surprising enhancement could be attributed to their ability to migrate over relatively long distances in soil, potentially including vertical movement from unsampled horizons (i.e., either from the organic layer above or the mineral layer below the horizon where the necromass bags were incubated) as they actively seek out ephemeral organic matter patches (Griffiths and Caul 1993). In accordance with our second hypothesis (H2), incubation time further shaped nematode OTU richness: it peaked early in decomposition before declining over time. Such a pattern aligns with well‐documented observations from plant residue studies, where nematode populations typically reach maximum densities early in decay and then decrease (Georgieva et al. 2005; Griffiths et al. 1993; Griffiths and Caul 1993; Lenz and Eisenbeis 1998; Wang et al. 2004; Wasilewska et al. 1981; Wasilewska 1992). In line with Christensen et al. (1992), we observed that nematodes in necromass were strongly enriched in bacterivore taxa relative to surrounding soil, particularly at early decay stages where almost all nematode communities were dominated by bacterivores. These results diverge from the expectation that the nematode communities would be co‐dominated by bacterivores and fungivores, given that fungal necromass hosts abundant bacterial and fungal decomposers in this study. Nonetheless, they are surprisingly consistent with well‐documented patterns in plant litter, where bacterivorous nematodes in Rhabditidae and Diplogastridae typically predominate early in the decay process (Christensen et al. 1992; Georgieva et al. 2005; Griffiths et al. 1993; Griffiths and Caul 1993; Lenz and Eisenbeis 1998; Wasilewska et al. 1981; Wasilewska 1992; Wang et al. 2004). In our study, the bacterivorous genera *Rhabditis*, *Mesorhabditis*, and *Choriorabditis* (Rhabditidae) as well as *Pristionchus* (Diplogastridae) were prominent early colonisers, whereas the fungivorous genus *Aphelenchoides* prevailed at later decay stages. Thus, it appears that the nematode composition on fungal necromass mirrors the successional trajectories observed in plant litter systems, characterised by an early increase of bacterivores that are subsequently replaced by fungivores. Collectively, these results provide the first direct evidence that nematodes actively colonise and respond to fungal necromass decay in a manner broadly parallel to plant debris, suggesting a shared ecological framework underlying nematode community dynamics in transient organic matter patches in soils.

### 4.4 Protist Community Dynamics During Fungal Necromass Decay

For protist communities, as with nematodes, most comparisons must be drawn from plant litter decomposition, as this is the first published data on protist assemblages in decomposing fungal necromass to our knowledge. In terms of taxonomy, our findings parallel prior plant litter‐based studies reporting Cercozoa and Lobosa lineages dominating decaying plant debris (Bonanomi et al. 2019; Voss et al. 2019). Unlike nematodes, however, protist OTU richness was consistently lower in necromass relative to soil, aligning with our first hypothesis (H1) and matching patterns observed for bacteria or fungi. Moreover, in agreement with our second hypothesis (H2), incubation time had an opposite effect on protists compared with nematodes: whereas nematode richness declined over time, protist richness increased. This result, however, also concurs with studies of plant litter, which have shown that protists often rise in abundance and diversity more gradually as decomposition advances (Bonanomi et al. 2019; Georgieva et al. 2005; Wang et al. 2004). Compared to soil, we found fungal necromass to be significantly enriched in primary consumer protists and depleted in parasites, matching patterns reported by Voss et al. (2019). However, regarding plant litter decay, numerous studies have described an early dominance of flagellate taxa primarily feeding on bacteria and a later prevalence of naked and testate amoebae with broader feeding ranges (Bonanomi et al. 2019; Georgieva et al. 2005; Griffiths et al. 1993; Griffiths 1994). Here, we observed only minimal shifts in protist community composition over time—its response was about three times smaller than that of nematodes. Further, while *Saccamoeba* was most abundant early in decomposition, certain flagellate genera (*Sandona*, *Cercomonas*, *Neocercomonas*, *Eocercomonas*) dominated at later stages.

Overall, these protist community results do not align with the succession patterns typically reported for plant litter, where bacterial‐feeding flagellates and, to a lesser extent, amoebae show clear temporal shifts. Instead, our data suggest that necromass‐associated protist assembly diverges from plant‐based systems, a contrast further underscored by the aforementioned nematode community dynamics. Interestingly, protist communities were more strongly influenced by necromass type, presumably because they are more specialised feeders on bacterial and fungal decomposers that are themselves highly responsive to melanin content. Relatively high prey specificity for protist predators has indeed been highlighted before (Fujino et al. 2024). In contrast, and in disagreement with our second hypothesis (H2), nematodes, possibly due to broader dietary ranges, were less sensitive to the biochemical variation of the necromass inducing changes in microbial decomposer assemblages. Taken together, it appears the assembly rules of protists colonising fungal necromass are fundamentally different than those present in plant‐based systems, with necromass biochemical composition exerting a stronger effect than decomposition time as the key factor driving protist community shifts. Finally, microbial predator communities within fungal necromass—both protists and nematodes—exhibit distinct diversity and composition compared to those in bulk soils, mirroring patterns observed for secondary decomposers. This divergence likely reflects the fact that necromass is an ephemeral resource patch, one that requires active colonisation from surrounding soils and sustains prey communities differing from bulk soil in both composition and abundance. Consequently, fungal necromass functions as a transient hotspot of decomposition and predation, governed by its own assembly rules that stand in sharp contrast to the nutrient‐poor bulk soil landscape.

### 4.5 Potential Indirect Effects of Microbial Predators on Fungal Necromass Decay

Angst et al. (2024) recently underscored the need to integrate predators into soil biogeochemical models, particularly to advance our understanding of SOM formation. Partially supporting our third hypothesis (H3), structural equation modelling based on an a priori framework of potential top‐down control of fungal necromass decomposition by microbial predators revealed that predator community composition significantly influenced both bacterial and fungal abundances, which in turn marginally (*p* = 0.05–0.10) influenced necromass mass loss. Specifically, we found that nematodes during the early stages of necromass decay and both nematodes and protists in the later stages appeared to be linked with the abundances of bacterial and fungal decomposers. This finding aligns with broader soil ecology research documenting tight connections between microbial predators and microbial decomposer biomass, diversity, and community structure (De Mesel et al. 2004; García‐Palacios et al. 2016; Ingham et al. 1985; Jiang et al. 2017, 2018; Kane et al. 2022; Liao et al. 2023; Mielke et al. 2022). Moreover, we found suggestive evidence, consistent across both necromass types, that shifts in microbial decomposer abundance caused by predators were associated with necromass mass loss, corroborating other studies showing that microbial predation often cascades into modified organic matter decomposition rates (García‐Palacios et al. 2016; Geisen et al. 2021; Jiang et al. 2018; Kane et al. 2022; Wang et al., 2024). Specifically, bacterial abundance was pivotal in explaining necromass mass loss, with putative modifications of bacterial abundances by nematodes affecting necromass mass loss only after 4 weeks of decomposition in low melanin necromass and by both nematodes and protists after 12 weeks in high melanin necromass, occurring just before stabilisation in mass loss. This carries significant implications for fungal necromass carbon stocks, given that the stabilised fraction is highly resistant to microbial breakdown. Additionally, the absence of indirect links between microbial predators and necromass decay during the active‐decay phase in our top‐down SEMs suggests that bottom‐up control of predator communities, that is, where shifts in secondary decomposer composition and abundance shape predator composition, may dominate necromass turnover when necromass quality and resource availability for decomposers are high (Lenoir et al. 2007). Taken together, these findings suggest that secondary decomposer processes in fungal necromass may be regulated by predator–decomposer interactions, with potential for knock‐on effects on decomposition rates. As such, our results further support calls for including microbial predator activities in research frameworks addressing fungal necromass decomposition and its contribution to SOM formation.

### 4.6 Limitations and Future Research Directions

It is important to note that our study design does have inherent limitations. First, our mesh‐bag approach excludes larger soil fauna, such as mites and collembola, which can feed on microbial predators or decomposers and could further alter necromass decay pathways (Vreeken‐Buijs and Brussaard 1996). Second, although we show that microbial predators are linked to microbial decomposer abundance and potentially indirectly mediate fungal necromass decomposition rates, our SEM models were relatively simple, whereas actual soil food webs are typically complex. For example, inter‐predator interactions alone can involve nematodes feeding on protists (Anderson et al. 1977), protists feeding on nematodes (Geisen et al. 2015), and fungi trapping nematodes (Drechsler 1933). Moreover, our use of functional‐group classifications (e.g., bacterivores vs. fungivores in nematodes) necessarily oversimplifies reality: many microbes have facultative feeding strategies and can expand their prey range under different environmental conditions, as exemplified by bacterivorous protists that also feed on fungi (Dumack et al. 2020; Geisen et al. 2016). Moreover, our test of potential indirect controls by microbial predators on necromass decay via a priori top‐down SEM models remains correlative. Future studies incorporating manipulative experiments will be instrumental in determining the actual top‐down or bottom‐up controls within the brown food web during fungal necromass decay (Lenoir et al. 2007; Shurin et al. 2012). Notwithstanding these constraints, our findings emphasise the importance of considering microbial predation in regulating fungal necromass associated microbial communities and their potential to indirectly influence necromass decomposition. Moving forward, controlled experiments in which both microbial decomposers and predators (micro‐ and mesofauna) are inoculated under fully factorial designs will be essential to predict more accurately how microbial predation influences the decay of fungal necromass (Geisen et al. 2021). Importantly, this is feasible because most bacteria and fungi constituting the core fungal necrobiome are readily cultured (Kennedy and Maillard 2023). Moreover, our study identified a range of protist and nematode taxa abundant during necromass decay, which can also be isolated or obtained from culture collections.

## 5 Conclusions

Here, we demonstrate that decomposing fungal necromass represents a transient microbial hotspot in the brown food web, whose architecture is co‐governed by necromass biochemical quality and decomposition stage, and is taxonomically and functionally distinct from the surrounding soil matrix. Both low and high quality fungal necromass support elevated bacterial and fungal abundances, with a relative dominance of fungi over bacteria compared to soil, and distinct microbial community compositions. Microbial predators colonising fungal necromass patches differ markedly from those in the soil matrix, likely due to environmental filters such as migration barriers and prey selection. These differences appear to be governed by contrasting drivers: protist communities are primarily shaped by necromass quality, likely reflecting specific within‐kingdom feeding preferences on microbial decomposers, whereas nematode communities shift mainly with decomposition stage, mirroring an inter‐kingdom shift in prey dominance from bacteria to fungi. Finally, structural equation modelling did link predator community composition indirectly to fungal necromass mass loss, suggesting that predator–decomposer interactions can influence fungal necromass decay rates. By revealing how substrate quality and temporal dynamics jointly structure secondary decomposer and microbial predator communities, our study extends the fungal necrobiome framework to higher trophic levels and highlights the need for future experiments and modelling efforts that treat predation and substrate traits as co‐drivers of soil organic matter formation.

## Author Contributions

F.M. and P.G.K. designed the study. The experiments were conducted by F.M., B.H.B., and P.G.K. F.M. wrote the manuscript with help from P.G.K. F.M., S.G., E.L., and P.G.K. analysed the data. All the authors interpreted the data and approved the manuscript draft.

## Conflicts of Interest

The authors declare no conflicts of interest.

## Supporting information


**Figure S1:** Accumulation curves of OTUs for bacterial, fungal, protist, and nematode communities in bulk soil and fungal necromass, differentiated by melanin content (high vs. low melanin necromass) and incubation time (4 vs. 12 weeks).

**Figure S2:** A priori conceptual structural equation model (SEM) illustrating the hypothesised pathways by which protist and nematode community structures may influence bacterial and fungal abundances, which in turn may affect fungal necromass mass loss. Arrows represent hypothesised causal effects between variables, and double‐headed arrows denote parameters that may covary in the model (i.e., protist and nematode communities, and bacterial and fungal abundances).

**Figure S3:** Remaining fungal necromass mass after 4 or 12 weeks of incubation, in necromass with low or high melanin content. Mixed‐effects models with ‘Plot’ as a random factor were followed by ANOVA. *p < 0.05, **p < 0.01, ***p < 0.001.

**Figure S4:** Mean relative abundances of (A) bacterial classes, (B) fungal classes, (C) protist phyla, and (D) nematode classes in necromass with low or high melanin content, incubated for 4 or 12 weeks, compared with bulk soil. The number of biological replicates for each treatment is listed in Table S1.

**Table S1:** Number of samples per habitat (soil or necromass) and necromass type (low or high melanin content) included in the final analysis.

**Table S2:** Good's coverage (mean ± SE) for bacterial, fungal, protistan and nematode communities. Values are reported for bulk soil and for fungal necromass depending on melanin content (high vs. low melanin necromass) and incubation time (4 or 12 weeks).

**Table S3:** Effects of sampling date on soil microbial parameters: bacterial, fungal, protist and nematode OTU richness and community composition, together with bacterial and fungal abundances estimated by qPCR. Differences in OTU richness and in bacterial and fungal abundances between sampling dates were analysed with paired *t*‐tests, whereas community composition was compared with PERMANOVA based on Bray–Curtis dissimilarity.

**Table S4:** Genera significantly influenced by habitat (soil or necromass) for bacteria, fungi, protists, and nematodes.

**Table S5:** Genera significantly influenced by necromass melanin content (low or high) for bacteria, fungi, protists, and nematodes.

**Table S6:** Genera significantly influenced by necromass incubation time (4 or 12 weeks) for bacteria, fungi, protists, and nematodes.

## Data Availability

All fastq files used for microbial community analysis, along with corresponding sample metadata, have been deposited in the NCBI Short Read Archive (accession numbers: bacteria (16S) PRJNA1231136, fungi (ITS) PRJNA1233824, and protists and nematodes (18S) PRJNA1233825), and additional data collected during this study have been deposited on Dryad, including necromass mass loss data, and microbial abundance data (https://datadryad.org/dataset/doi:10.5061/dryad.x3ffbg7ws). Benefits from this research accrue from the sharing of our microbial community sequencing data and associated metadata on public databases as described above.
