# Supplementary figures and images for: Secondary Decomposers Meet Their Predators: Decomposition Stage and Substrate Quality Jointly Structure Microbial Brown Food Webs During Fungal Necromass Decay
Source: Mol Ecol. 2025 Aug 8;34(18):e70060. doi: 10.1111/mec.70060 (PMC12421484; doi:10.1111/mec.70060)

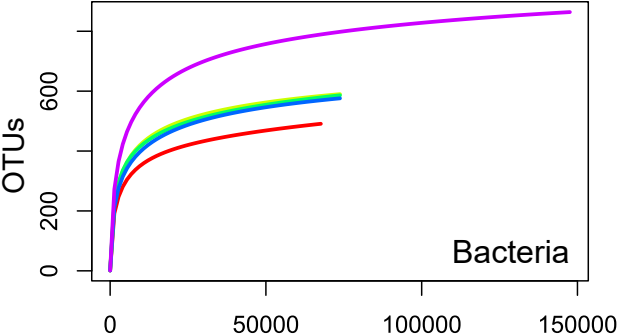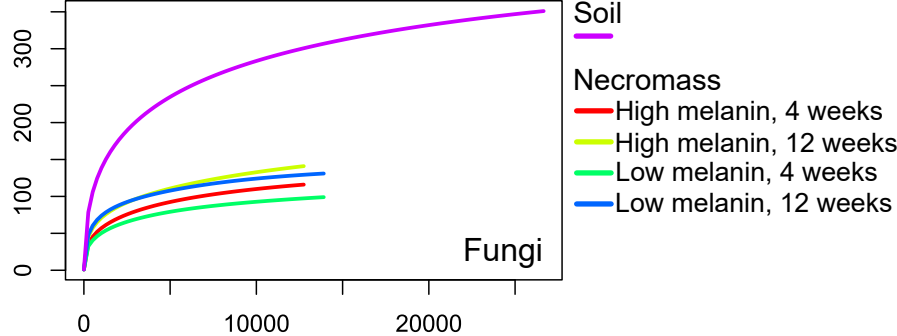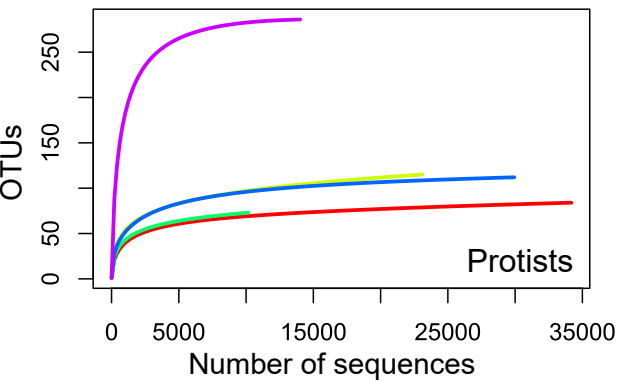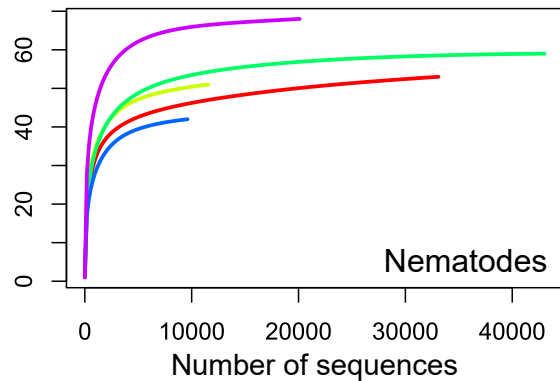

Supplement: Supplementary file 1 — Figure S1: Accumulation curves of OTUs for bacterial, fungal, protist, and nematode communities in bulk soil and fungal necromass, differentiated by melanin content (high vs. low melanin necromass) and incubation time (4 vs. 12 weeks). [file MEC-34-e70060-s009.pdf]

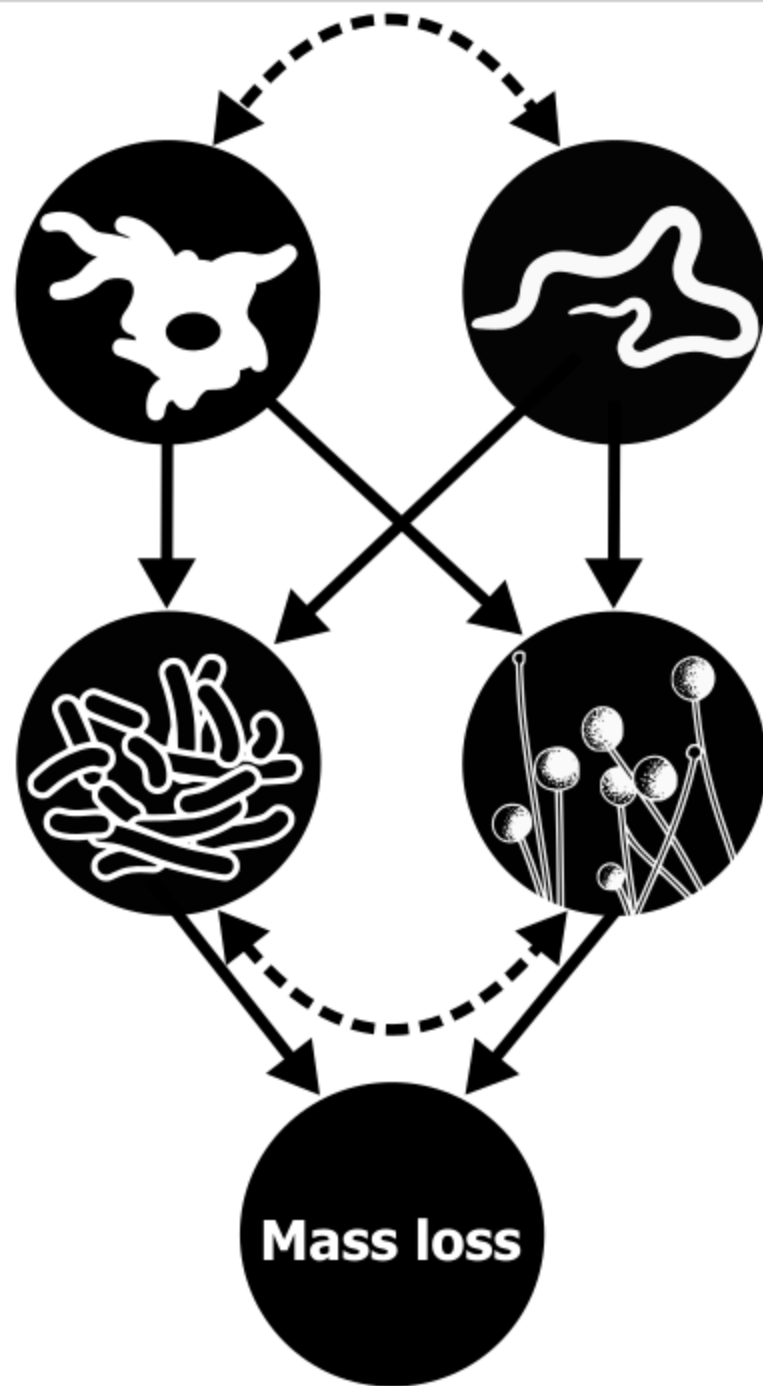

## Community composition

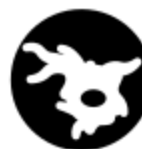

Protists

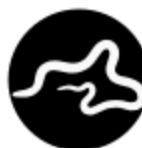

Nematodes

## Abundance

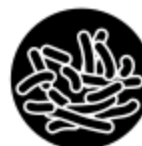

Bacteria

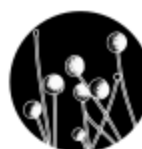

Fungi

Supplement: Supplementary file 2 — Figure S2: A priori conceptual structural equation model (SEM) illustrating the hypothesised pathways by which protist and nematode community structures may influence bacterial and fungal abundances, which in turn may affect fungal necromass mass loss. Arrows represent hypothesised causal effects between variables, and double‐headed arrows denote parameters that may covary in the model (i.e., protist and nematode communities, and bacterial and fungal abundances). [file MEC-34-e70060-s004.pdf]

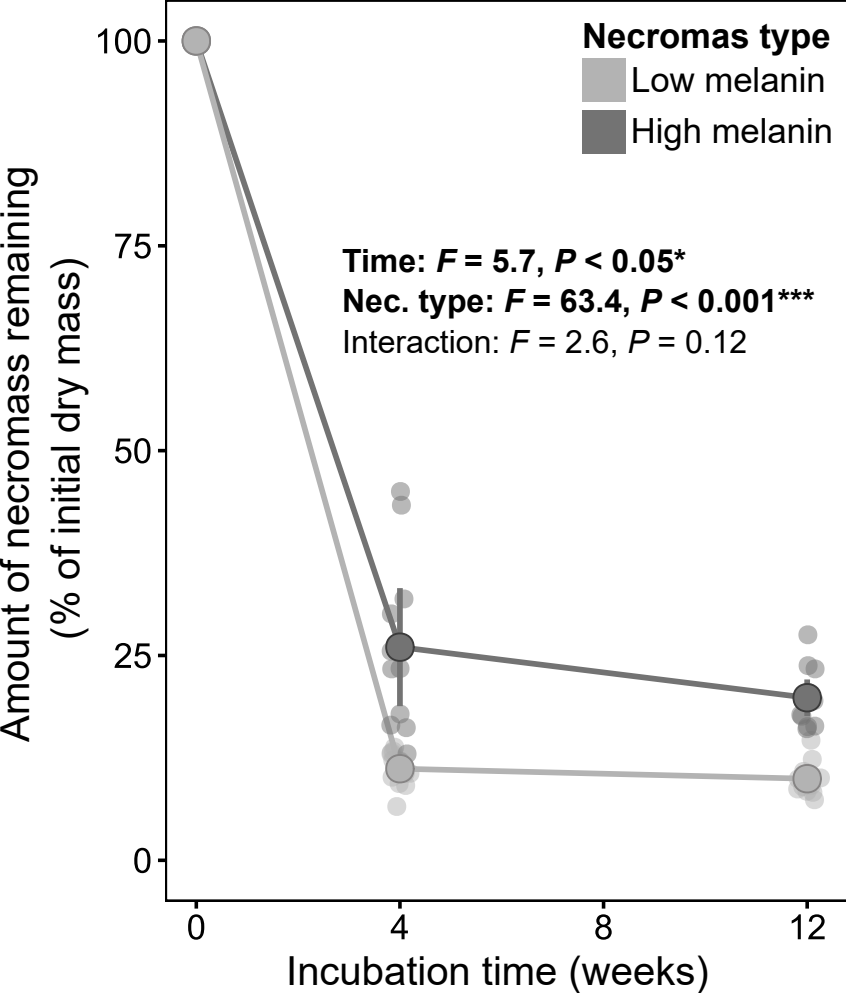

Supplement: Supplementary file 3 — Figure S3: Remaining fungal necromass mass after 4 or 12 weeks of incubation, in necromass with low or high melanin content. Mixed‐effects models with ‘Plot’ as a random factor were followed by ANOVA. *p < 0.05, **p < 0.01, ***p < 0.001. [file MEC-34-e70060-s003.pdf]

(A)

**Bacteria**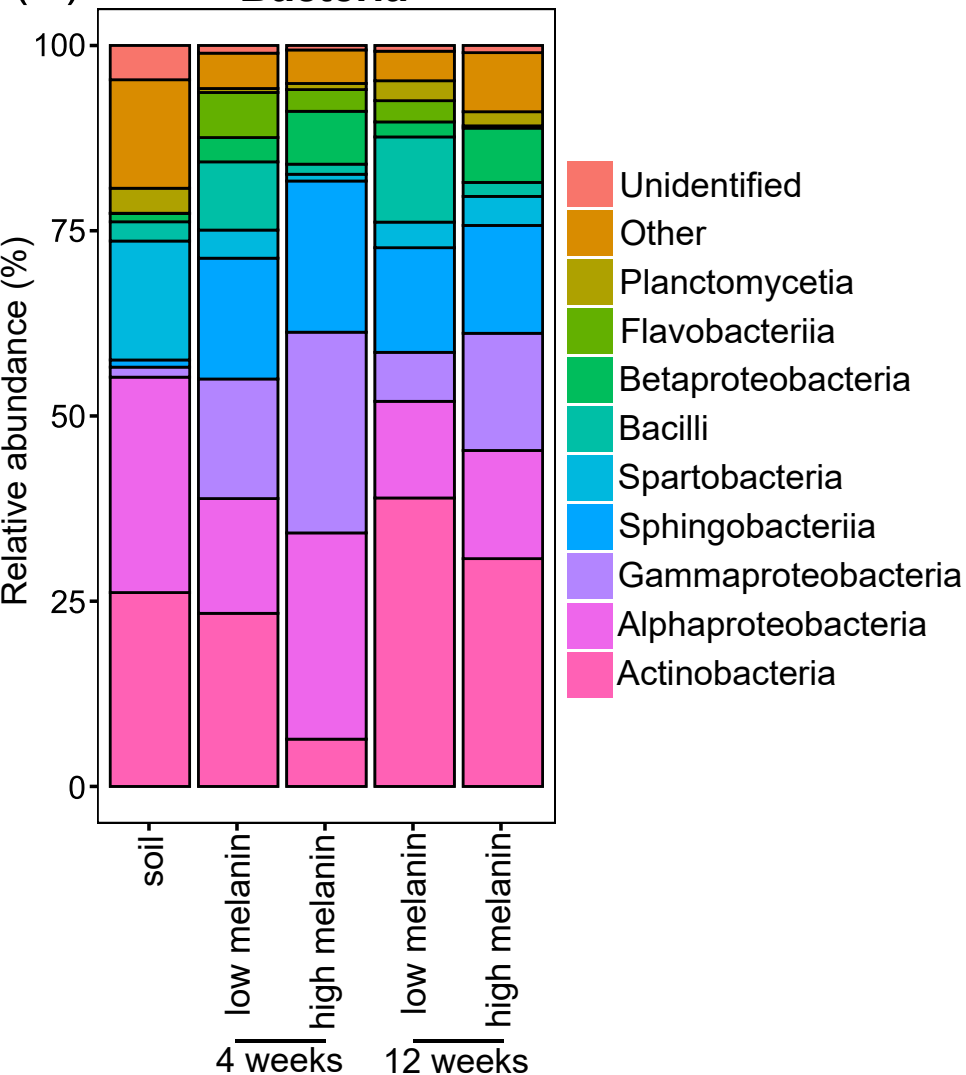

(B)

**Fungi**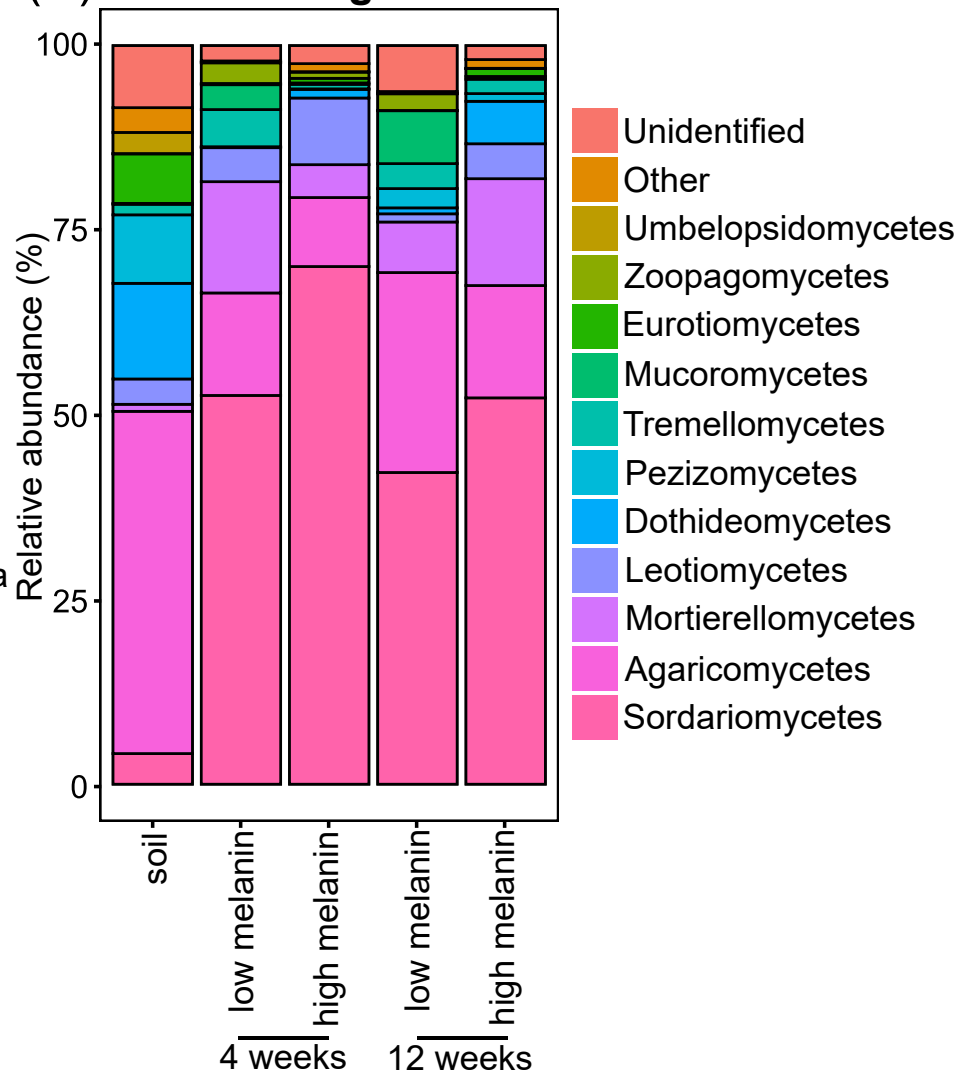

(C)

**Protists**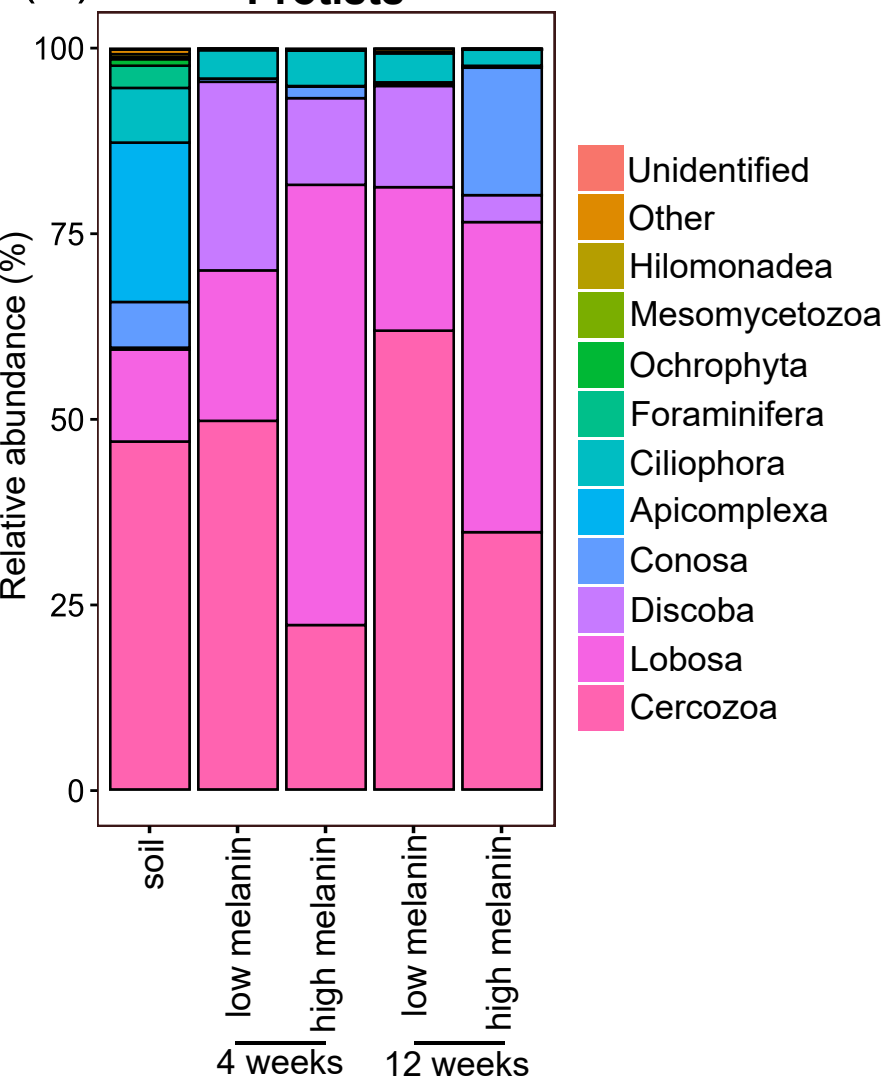

(D)

**Nematodes**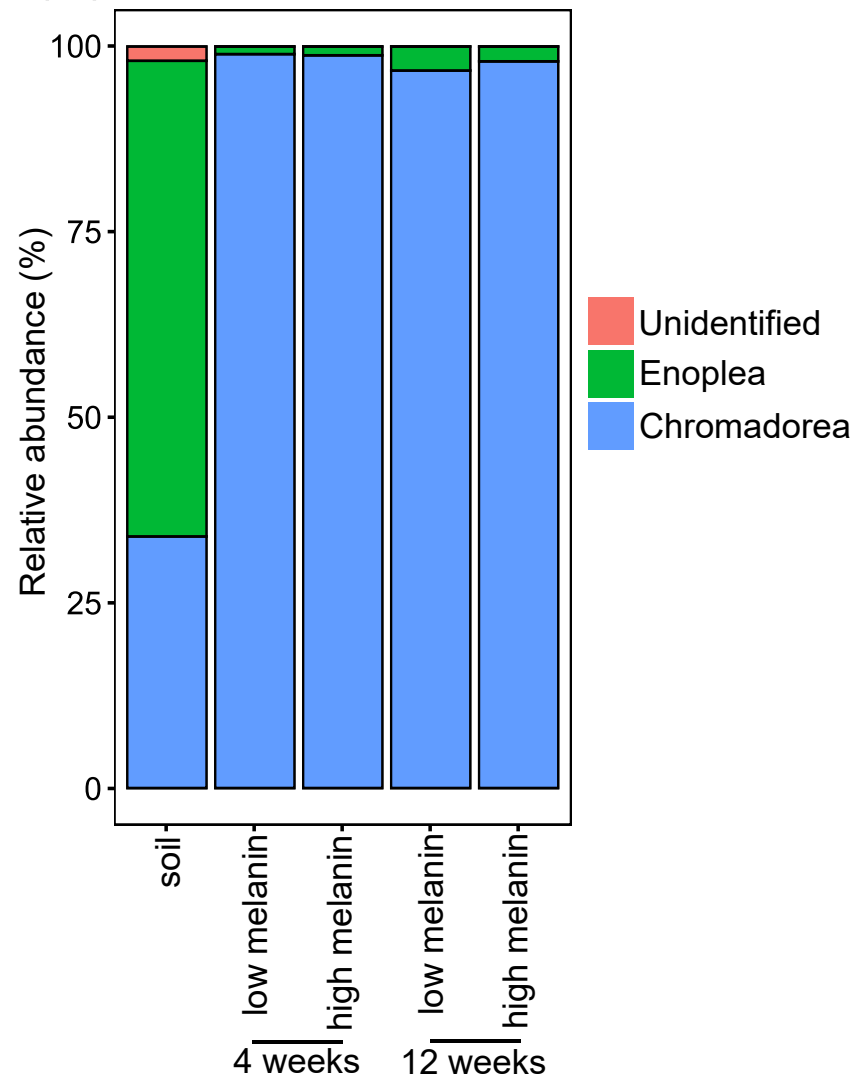

Supplement: Supplementary file 4 — Figure S4: Mean relative abundances of (A) bacterial classes, (B) fungal classes, (C) protist phyla, and (D) nematode classes in necromass with low or high melanin content, incubated for 4 or 12 weeks, compared with bulk soil. The number of biological replicates for each treatment is listed in Table S1. [file MEC-34-e70060-s005.pdf]
